# Fish-otoliths from the marine-brackish water transition from the Middle Miocene of the Belgrade area, Serbia

**DOI:** 10.1007/s12542-015-0272-6

**Published:** 2015-07-24

**Authors:** Werner Schwarzhans, Katarina Bradić, Ljupko Rundić

**Affiliations:** 1 Ahrensburger Weg 103, 22359 Hamburg, Germany; 2 Natural History Museum of Denmark, Zoological Museum, Universitetsparken 15, 2100 Copenhagen, Denmark; 3 Department of Paleontology, Faculty of Mining and Geology, University of Belgrade, Kamenička 6, 11000 Belgrade, Serbia; 4 Department of Regional Geology, Faculty of Mining and Geology, University of Belgrade, Kamenička 6, 11000 Belgrade, Serbia

**Keywords:** Middle Miocene, Otoliths, Gobiidae, Systematics, Paleoecology, Mittel-Miozän, Otolithen, Gobiidae, Systematic, Paläoökologie

## Abstract

We describe here the first fossil otoliths from the Middle Miocene (Badenian and Sarmatian) of Belgrade, Serbia. They were obtained from Lower Badenian outcrops at Slanci and from upper Badenian and Sarmatian sediments recovered from four shallow wells near the village of Barajevo. The otoliths from the Lower Badenian of Slanci represent fishes typical for an open marine environment, characterized primarily by the mesopelagic families Myctophidae and Bregmacerotidae, a faunal composition that is also well known from other time equivalent locations in the Central Paratethys. The upper Badenian and Sarmatian composition of the fish fauna, in contrast, is dominated by otoliths of the family Gobiidae, indicating a sharp environmental shift from open marine to shallow water, probably slightly brackish environments, which is also confirmed by the faunal composition of mollusks, foraminifera, and ostracods. Most of the gobiid genera identified in the samples from Barajevo represent small fishes of the so-called sand gobies with Ponto-Caspian affinities, such as *Economidichthys, Knipowitschia*, or *Pomatoschistus*, or are entirely endemic to the Ponto-Caspian Basin, such as *Hyrcanogobius*. Another group of endemic Ponto-Caspian gobies is the first fossil record interpreted to represent the genus *Proterorhinus.* These and other finds currently being investigated indicate that the origin of the extant, rich, endemic gobiid fauna of the Ponto-Caspian Basin dates back to a crucial time in the development of Paratethys during the Middle Miocene when it segregated from the Mediterranean with the onset of phases of low salinity in the basin. In addition, we briefly discuss the distribution of certain gobiid species during Late Badenian and Sarmatian as it begins to emerge. The following new taxa are described based on fossil otoliths: *Hyrcanogobius hesperis* n.sp. and *Proterorhinus vasilievae* n.sp.

## Kurzfassung

Es werden die ersten fossilen Otolithen aus dem Mittel-Miozän (Badenien und Sarmatien) von Belgrad, Serbien beschrieben. Sie stammen von Aufschlüssen des unteren Badenien nahe Slanci und aus vier Bohrungen nahe Barajevo, in denen oberes Badenien aund Sarmatien durchteuft wurden. Die Otolithen aus dem unteren Badenien von Slanci repräsentieren Fische des offenen marinen Environments, primär charakterisiert durch die mesopelagischen Familien Myctophidae und Bregmacerotidae, eine Faunenassoziation wie sie aus anderen zeitgleichen Lokationen der Zentralen Paratethys gut bekannt ist. Im Gegensatz dazu wird die Fischfauna des oberen Badenien und des Sarmatien dominiert von Otolithen der Familie Gobiidae, die somit einen scharfen ökologischen Wechsel von offen marin zu Flachwasser, möglicherweise sogar Brackwasser, anzeigen, was durch Untersuchungen an Mollusken, Foraminiferen und Ostracoden bestätigt wird. Die meisten der nachgewiesenen Gobiiden-Gattungen in den Proben von Barajevo stammen von den sogenannten „Sand-Grundeln“, die auch heute im Ponto-Kaspischen Raum häufig anzutreffen sind, wie zum Beispiel *Economidichthys, Knipowitschia* und *Pomatoschistus* oder sogar endemisch sind, wie *Hyrcanogobius.* Eine weitere Gruppe endemischer Ponto-Kaspischer Gobiiden ist durch Otolithen der Gattung *Proterorhinus* vertreten. Zusammen mit anderen, zur Zeit in Bearbeitung befindlichen Funden aus der Paratethys wird deutlich, dass die heutige, reiche, endemische Gobiiden-Fauna des Ponto-Kaspischen Beckens bis in einen Schlüssel-Zeitraum der Entwicklung der Paratethys im Mittel-Miozän zurückreicht, in der sie vom Mittelmeer zunehmend abgetrennt wurde und Phasen reduzierter Salinität einsetzten. Des weiteren wird das sich langsam ergebende Bild der Verbreitung der verschiedenen Gobiiden-Arten im oberen Badenien und Sarmatien der Paratethys kurz umrissen. Zwei neue auf Otolithen basierende Arten werden beschrieben: *Hyrcanogobius hesperis* n.sp. und *Proterorhinus vasilievae* n.sp.

## Introduction

During the Middle Miocene, the Paratethys, a once continuous marine water body stretching from southern Germany and Austria in the west beyond the Caspian Sea and Aral Lake in the east, began to segregate into several basins and subbasins with varying environments giving rise to the development of a complex and interacting endemic evolution of its biota (Rögl and Steininger 1983; Rögl 1998; Popov et al. 2006). Nowadays, the Caspian Sea and Black Sea with its freshwater distributaries constitute the highly endemic Ponto-Caspian bioprovince, the “survivors” of a chiefly Eastern Paratethyan paleo-bioprovince of earlier Neogene times. In teleosts, these endemic “survivors” are mostly species of the Gobiidae, a fish family particularly well adapted to environments in the marginal marine to freshwater interfaces (Miller 2003, 2004). The status of the knowledge base of teleost records, primarily otoliths, in the Neogene of the Eastern Paratethys has been extensively discussed in Bratishko et al. (2015).

Contrary to the history in the Eastern Paratethys, there is no obvious link of a former Central Paratethyan fish fauna to an extant bioprovince. Few endemic and exclusively freshwater gobiids found in northern Italy, along the Balkans and in Greece have been related to “Lago Mare” events in the early Pliocene (Miller 2004), but may have earlier roots, as will be discussed later. The knowledge of fossil teleost otoliths in the Central Paratethys is generally good in the fully marine strata of the Lower Badenian, and has revealed a rich and diverse fauna. Radwanska (1992) recorded 145 otolith-based taxa from the Badenian of Poland. The well-studied faunas from the northwestern Central Paratethys are similarly rich as documented by Brzobohaty (1994, 1995), Brzobohaty et al. (2007), Brzobohaty and Nolf (2002), Nolf and Brzobohaty (2009), Prochazka (1893), and Schubert (1902, 1905, 1906, 1912). A smaller, but distinctly diverse early Badenian otolith assemblage was described by Weiler (1950) from Costeiul-De-Sus (Kostej) in the Banat Basin of Romania. Our knowledge of Late Badenian to Sarmatian otolith associations in the Central Paratethys is much more sparsely distributed. The Late Badenian of the Pannonian Basin has yielded a still-rich marine otolith-based fish fauna (Holec 1975; Schubert 1906, 1912; Bratishko et al. 2015), but the associations of the Pre-Carpathian Trough (Smigielska 1966; Brzobohaty 1980) and the Transylvanian Basin (Weiler 1943, 1949, 1950) document a restricted, partly endemic fish fauna following re-colonization after the Middle Badenian evaporitic crisis (Peryt 2006, 2013). Sarmatian otolith associations from the Central Paratethys are even sparser, widely apart, and with few species (Brzobohaty and Stancu 1974).

Here we describe the first otolith association from the Badenian and Sarmatian from the Belgrade city area, Serbia. Albeit a small faunula, it nevertheless represents yet another piece in the puzzle of reconstructing the intricate development of the fish fauna of the marine-brackish transition in the Paratethys. It also increases our recognition and understanding of the regional diversity in a strongly segmented water body.

## Regional geology

**Fig. 1:**
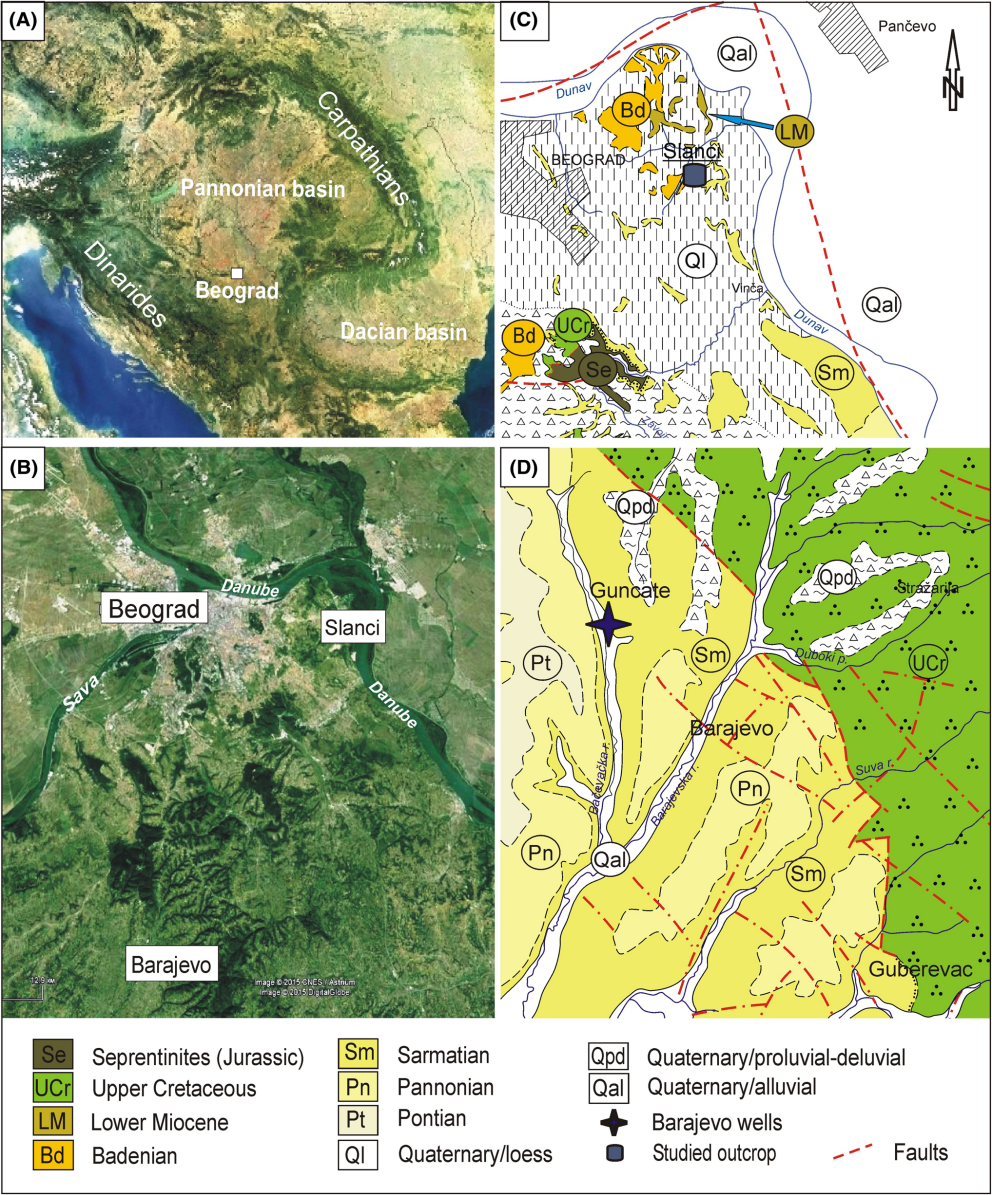
*A*. Satellite image of the Pannonian Basin and the Belgrade area (NASA, 2003); *B*. Position of the studied sites (Google Earth, 2015); *C*. Location of the Slanci outcrop from the Basic Geological Map (BGM), sheet Pančevo 1:100, 000 (after Ivković et al. 1966–simplified); *D*. Position of the studied boreholes near Barajevo according to the Basic Geological Map (BGM), sheet Obrenovac 1:100,000 (after Filipović et al. 1979; Filipović and Rodin 1980)

The Middle Miocene at the city of Belgrade and its vicinity (Fig. 1) directly overlies rocks of Late Cretaceous or Lower Miocene sediments, respectively. Both Badenian and Sarmatian stages are well developed and have been extensively studied since the last century with a few pioneer observations dating from the nineteenth century. The Badenian shows a typical marine character (marine Badenian), while the Sarmatian exhibits a more brackish development. Many articles deal with the geology of the deposits in the Belgrade area, the most recent ones being by Knežević and Šumar (1993, 1994), Mitrović (1998); Mitrović and Rundić (1991); Mitrović et al. (2006); Petrović (1985, 1995); Petrović and Šumar (1990); Rundić and Mitrović (1995); Rundić et al. (1997, 2012). Detailed biostratigraphic analyses have been performed based primarily on the mollusk fauna, as well as foraminifera and ostracods (Džodžo-Tomić 1953, 1958; Gagić 1967, 1974; Laskarev 1924, 1950; Laskarev et al. 1931; Luković 1922; Pavlović 1890, 1911, 1922; Petrović 1962, 1970; Stevanović 1959, 1970, 1977; Veljković-Zajec 1953a, b). Fish skeletons have been described by Andjelković (1964, 1966, 1969, 1989).

## Badenian

Marine sediments of the Badenian are widely distributed in the Belgrade city area and its vicinity and exhibit a rather uniform facies setting. This is seen as the result of the marine Middle Miocene transgression in the Paratethys, flooding this area for the first time (Rundić et al. 2013). Outcrop sites at Kalemegdan, Višnjica, Tašmajdan, Slanci, and others have been known for a long time (e.g. Pavlović 1890, 1922; Laskarev 1924; Luković 1992; etc.), including several lithological units such as the Rakovica Sand, the Višnjica Clay, and Tašmajdan and Kalemegdan biogenic limestones (Leitha Limestone equivalent) (Fig. 1).

**Fig. 2:**
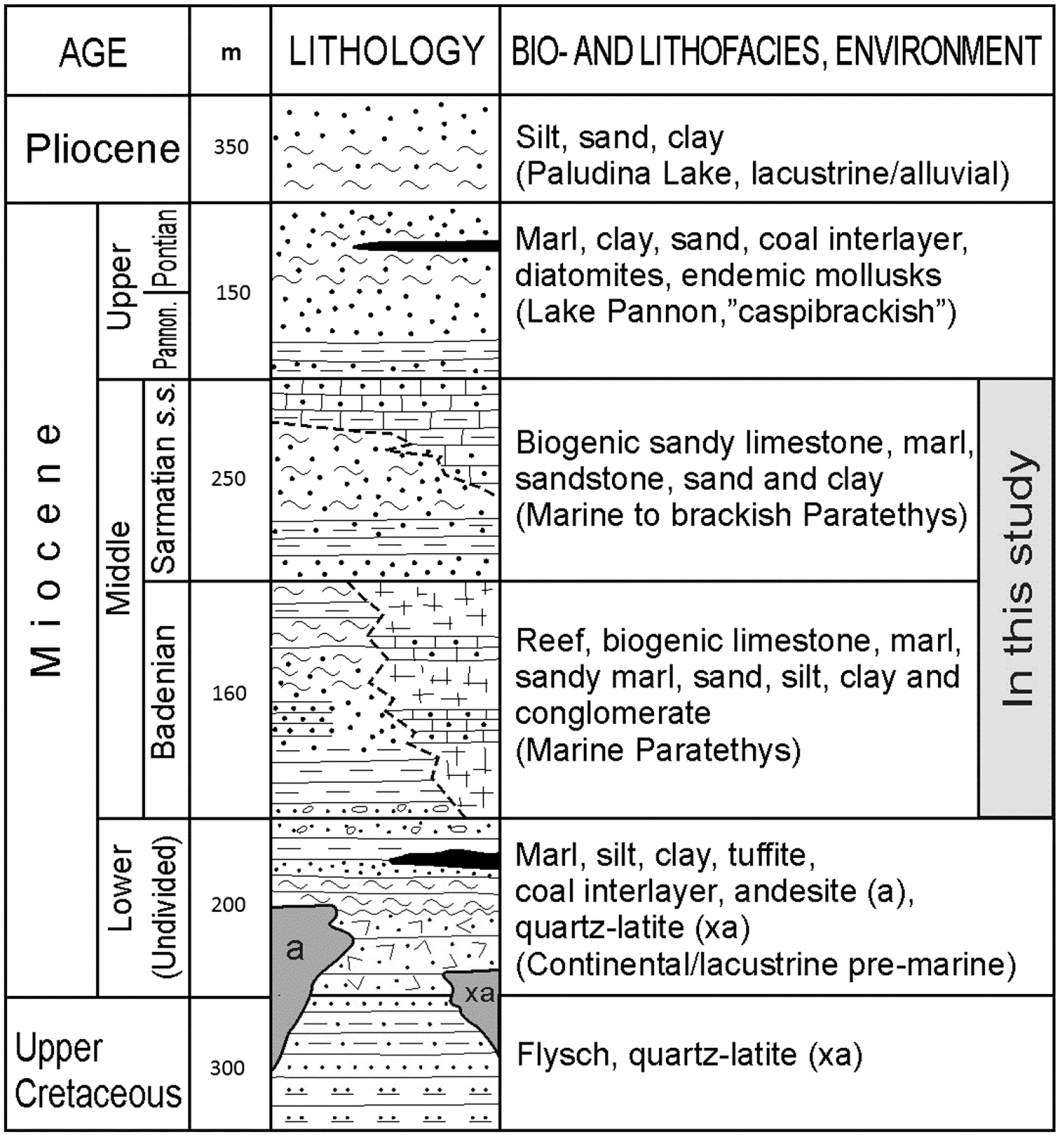
Synthetic lithostratigraphic column of the Neogene sequence of the Belgrade city area with average thickness. A grey rectangle outlines the stratigraphic range of the studied sediments (after the Basic Geological Maps 1:100,000, sheets Pančevo and Obrenovac)

A rich fossil fauna is mainly represented by different shallow marine organisms such as echinoids, bryozoans, corals, red algal assemblages, benthic foraminifera, ostracods, bivalves, and gastropods. However, deep-water faunal elements occur as well (e.g. rare cephalopods such as *Aturia aturi*, planktonic pteropods and foraminifera, ostracodes). Based on foraminifera, the Badenian of the Belgrade city area is divided onto three parts: the Lower Badenian Lagenidae Zone, the Middle Badenian *Spirorutilus carinatus* Zone, and the Upper Badenian *Elphidium crispum* and *Ammonia beccarii* Zone (Petrović 1985; Petrović and Šumar 1990) (Fig. 2).

**Fig. 3:**
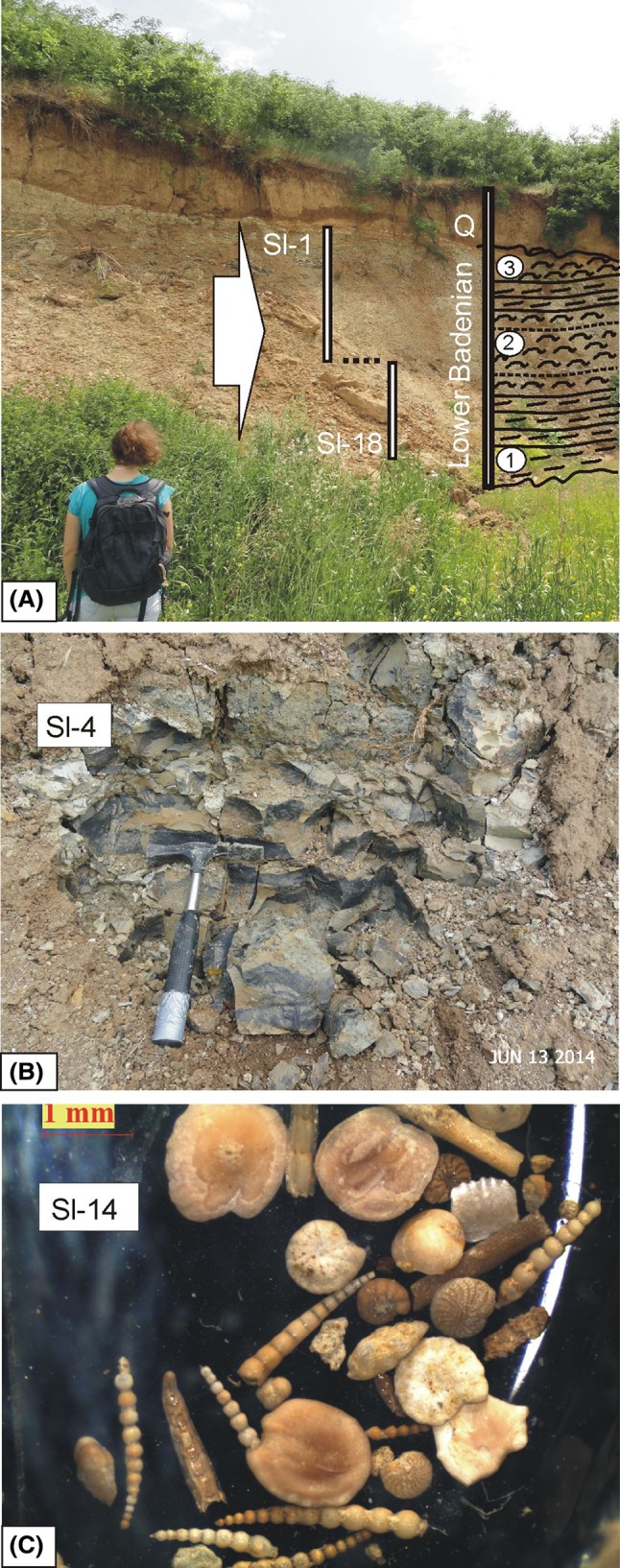
Lower Badenian sediments in the old clay pit near the village of Slanci; **a** Studied section indicating sample locations (*Sl 1–18*) and stratigraphic levels: *1* grey, massive, or low-bedded marls, *2* dark sandy clays, *3* yellow silt- and sandstones; *Q*–Quaternary; **b** Dark-blue sandy clays of sample *Sl-4*; **c** Detail of microfossil association of the Lower Badenian with foraminifera, ostracods, and otoliths (*Sl-14*)

*Slanci*: Otoliths were obtained from an outcrop section of up to 10 m height of Badenian sediments in the abandoned clay pit near the village of Slanci (Fig. 3—N 44°48′06.99″, E 20°35′32.98″). The section is composed of dark-gray, poorly bedded clays to massive marls, with thin silt and sand interlayers near the top (Fig. 3a). Macrofossils contain a low-diversity gastropod and pelecypod association (*Nucula nucleus, Anadara* sp., etc.) and rare fish and plant remains. The microfauna is rich in planktonic and benthic foraminifera, calcareous nannoplankton, ostracods, and sporadic otoliths. Biostratigraphically, it corresponds to the Lower Badenian, Lagenidae Zone (article in progress). Certain key planktonic foraminifera indicating the Upper Lageniade Zone are *Globigerinoides trilobus* and *Orbulina suturalis* (Petrović 1985). Furthermore, numerous and diverse benthic foraminifera and ostracoda correspond to this biostratigraphic level (e.g. *Uvigerina pygmaea, Bolivina dilatata, Bulimina elongata, Guttulina austriaca, Lagena* sp., *Cibicides* sp., *Amphistegina* sp., *Acanthocythereis hystrix, Parakrithe* cf. *crystallina, Bairdopillata subdeltoidea, Cnestocythere lamellicostata, Pterygocythereis calcarata, Henryhowella asperrima*).

**Fig. 4:**
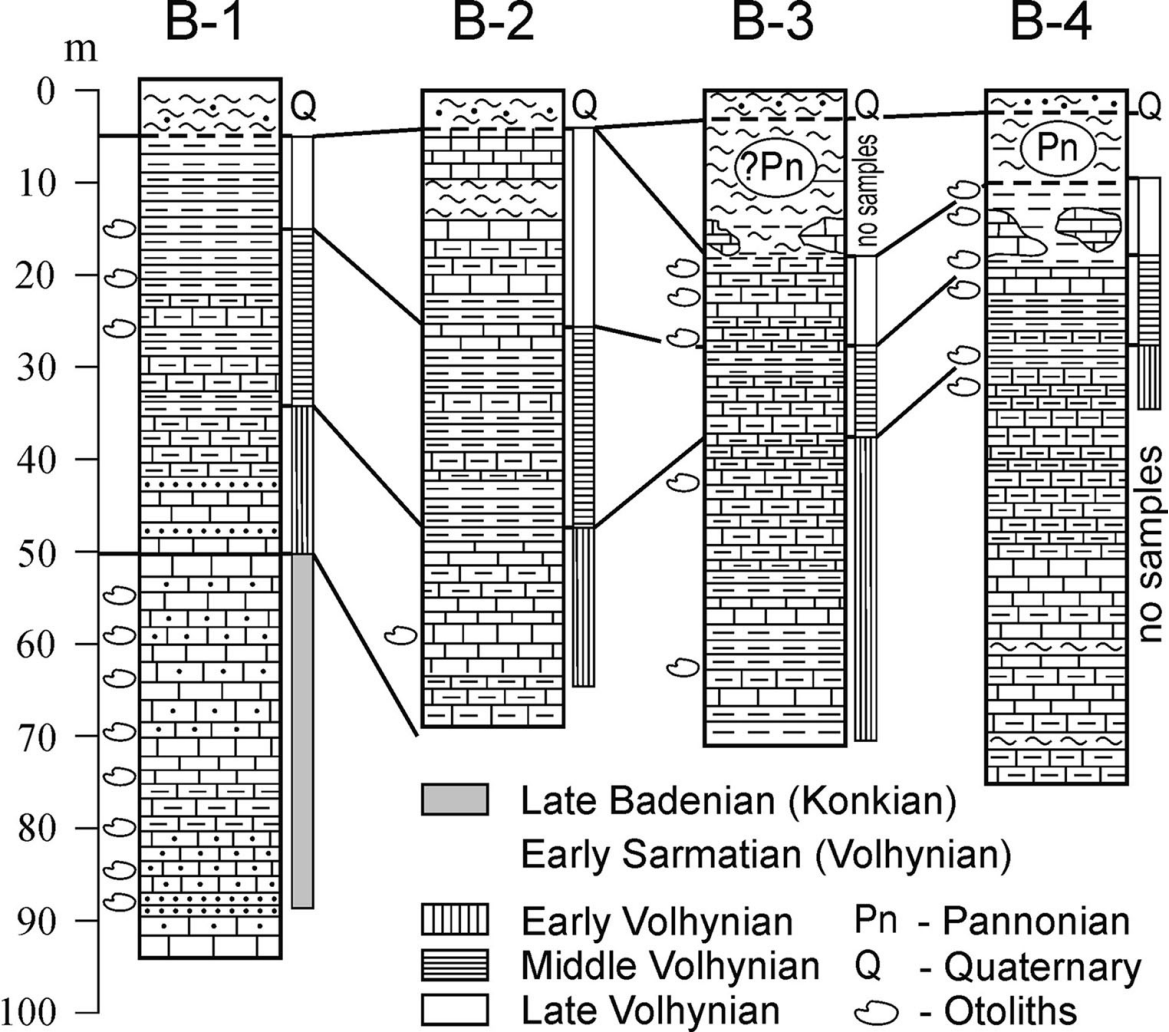
Lithostratigraphic correlation of the Barajevo wells (after Rundić and Mitrović 1995, and Mitrović 1998—modified). For lithology legend, see Fig. 2

*Barajevo*: Four hydrogeological wells were drilled near the southern limit of the municipal of Barajevo (village of Guncate), namely Barajevo B-1 (N 44°35′22.35″, E 20°23′37.84″), B-2 (N 44°35′11.18″, E 20°23′46.39″), B-3 (N 44°36′34.29″, E 20°22′48.78″), and B-4 (N 44°36′16.25″, E 20°23′03.85″) (Rundić and Mitrović 1995; Mitrović 1998). One of them, Barajevo B-1, found late Badenian sediments in the bottom section at a depth of 55–93 m (Fig. 4). Otoliths were obtained from marly and sandy limestones and sandstones, which cover the Upper Cretaceous sediments and represent the footwall for the Lower Sarmatian clastic-carbonate deposits. The following mollusks were identified by the mentioned authors: *Pecten* sp., *Venus* sp., *Arca* sp., *Hydrobia* sp., and *Cardium* sp. An abundant and rich microfauna indicates the uppermost part of the Badenian, the *Ammonia beccarii* Zone (Mitrović 1998).

## Sarmatian

Sarmatian deposits are also found widespread within the city limits of Belgrade and its vicinity (Knežević and Šumar 1993, 1994; Mitrović and Rundić 1991; Rundić and Mitrović 1995; Rundić et al. 1997, 2012; Šumar and Rundić 1992). They are composed of two main lithological units of brackish sediments: an older unit of early Sarmatian age (early and middle Volhynian) of mostly clastic sediments, and a younger unit of late Volhynian to early Bessarabian age represented by marls, sandy limestones, and massive, biogenic limestones. The older siliciclastic deposits are mainly developed in the northeastern part of the city area (Mirjevo, Lešće, Vinča, Ritopek, and Leštane), while the younger limestone level shows wider distribution further to the south (Čukarica, Rakovica, Leštane, Sremčica, Barajevo). The younger unit is incomplete in the wells Barajevo B-3 and B-4 with the top section eroded and hence the early Bessarabian equivalent likely missing.

*Barajevo*: In *Barajevo B-1* (Fig. 4) Sarmatian sediments were found from 5 to 50 m of depth. The section is composed of marl and marly limestone with interbeds of sandstone and was found relatively rich with foraminifera and ostracods. Biostratigraphically, the deposits were divided into three biozones (Rundić and Mitrović 1995): the deepest zone, characterized by large *Elphidium* specimens from 50 to 35 m, did not contain otoliths. The *Elphidium hauerinum* Zone (middle Volhynian) has been identified from 35 to 15 m and contained few otoliths. No otoliths were found in the youngest section of the *Porosononion granosum* Zone that extends from 15 to 5 m.

*Barajevo B-2* (Fig. 4) had lower Sarmatian marls and marly limestones in the depth interval from 62 to 4.50 m, again with the same subdivision as in Barajevo B-1. The zone with large *Elphidium* specimens (early Volhynian) from 62 to 47 m contained a single otolith. No otoliths were retrieved from the *Elphidium hauerinum* Zone at a depth of 47–25 m and the *Porosononion granosum* Zone at a depth of 25–4.50 m.

In *Barajevo B-3*, Sarmatian deposits were found at a depth of 70–19 m (Fig. 4), consisting of marls, marly limestones, and limestones. According to Mitrović (1998) the section is divided into three foraminiferal biozones: the *Elphidium reginum*, the *Elphidium hauerinum,* and the *Porosononion granosum* zones. The *Elphidium reginum* Zone (early Volhynian) was identified at a depth of 70–37 m and contained two otoliths. The *Elphidium hauerinum* Zone from 37 to 28 m contained no otoliths, and the *Porosononion granosum* Zone (Late Volhynian) from 28 to 19 m again contained a few otoliths.

In *Barajevo B-4*, Sarmatian sediments were found between 75 and 12 m (possibly to 10 m) (Fig. 4). However, only the last 36 m of the section was sampled (Mitrović, 1998). Three biozones of Volhynian age were identified: the early Volhynian *Elphidium reginum* Zone at a depth of 36–30 m, the middle Volhynian (*El. hauerinum* Zone) between 30 and 18 m, and the late Volhynian *Porosononion granosum* Zone from 18 to 12 (possibly to 10 m). All intervals contained a few otoliths. The last 10 m of the section belong to the Upper Miocene Pannonian silt and clay (without otoliths).

## Materials and methods

The otolith-bearing samples were collected in the field (Slanci) and from drilling cuttings of wells drilled (Barajevo B-1 to B-4) and washed and picked at the laboratory in Belgrade. A total of 73 otoliths were obtained.

The terminology for the morphological description of the otoliths follows Koken (1891), Weiler (1942), and Schwarzhans (1978). The morphometric measurements follow Schwarzhans (2013). The following abbreviations are used: otolith length = OL; otolith height = OH; otolith thickness = OT; sulcus length = SuL. For otoliths of Gobiidae, the curvature index of the inner face is calculated as percentage of OL.

Photos of otoliths were taken with a Wild M 400 photomacroscope at the location of the senior author and the photograph of *Diaphus acutirostrum* was taken at the laboratory at Belgrade. Individual multifocus photographs were processed with the Helicon Focus software by Helicon Soft for achieving a single continuous focused picture. Resulting photographs were digitally retouched as far as possible without afflicting alterations to the morphology of the shown otolith. For optimal comparison, all figures show otoliths from the right side, except for the otolith of the pleuronectiform *Arnoglossus*? *tenuis*, a species which depicts lateral dimorphism. Photos of left otoliths have, therefore, been mirrored and are marked accordingly in the figure captions. All photos show otoliths from the inner face, if not annotated differently.

The otoliths are deposited in the collection of the Chair of Historical Geology, Department of Regional geology, Faculty of Mining and Geology, University of Belgrade under the collection registration IGOTSL1-18 (Slanci) and IGOTBAB1-4 (Barajevo).

## Systematic paleontology

### Remarks

The otolith specimens from the early Badenian contain mostly species that are well known from the Miocene of the Paratethys, while those from the late Badenian and Sarmatian represent new or poorly known species. We have, therefore, reduced the descriptive part mostly to an annotated tabulation (Table 1) and focused on the new and rare findings, which mostly relate to the family Gobiidae. Since many of the contained gobiid otoliths show close relationship to extant Ponto-Caspian species we have also figured several Recent otoliths primarily of small gobiid species, from which otoliths were not or are poorly known before.

**Table 1:**
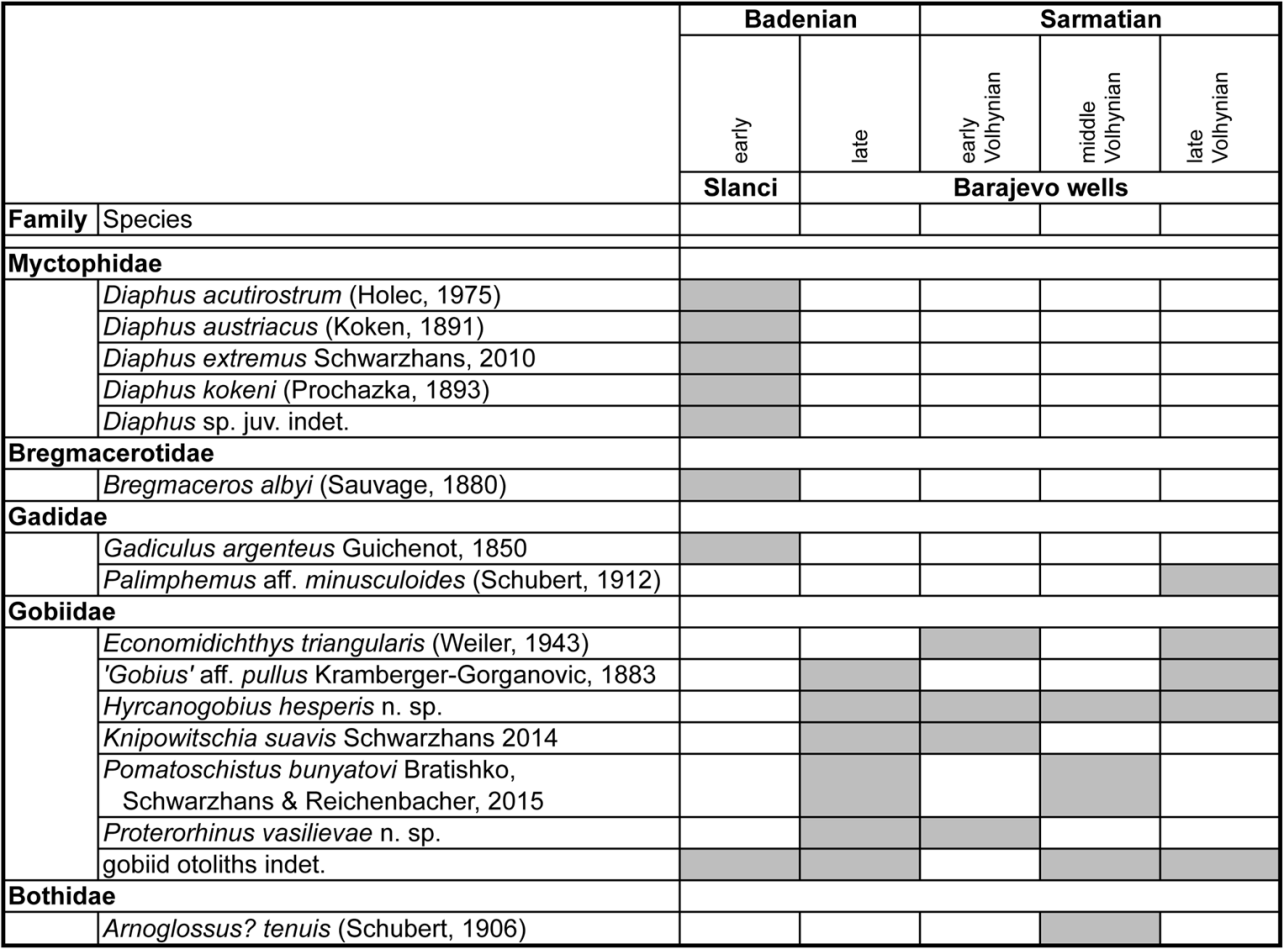
Stratigraphic distribution of obtained otoliths

The classification of the systematic part follows Nelson (2006).

Class Osteichthyes Huxley, 1880

Subclass Actinopterygii Klein, 1885

Order Myctophiformes Regan, 1911

Family Myctophidae Gill, 1893

Genus *Diaphus* Eigenmann and Eigemann, 1890

*Diaphus acutirostrum* (Holec 1975) (Fig. 5.4)

**Fig. 5:**
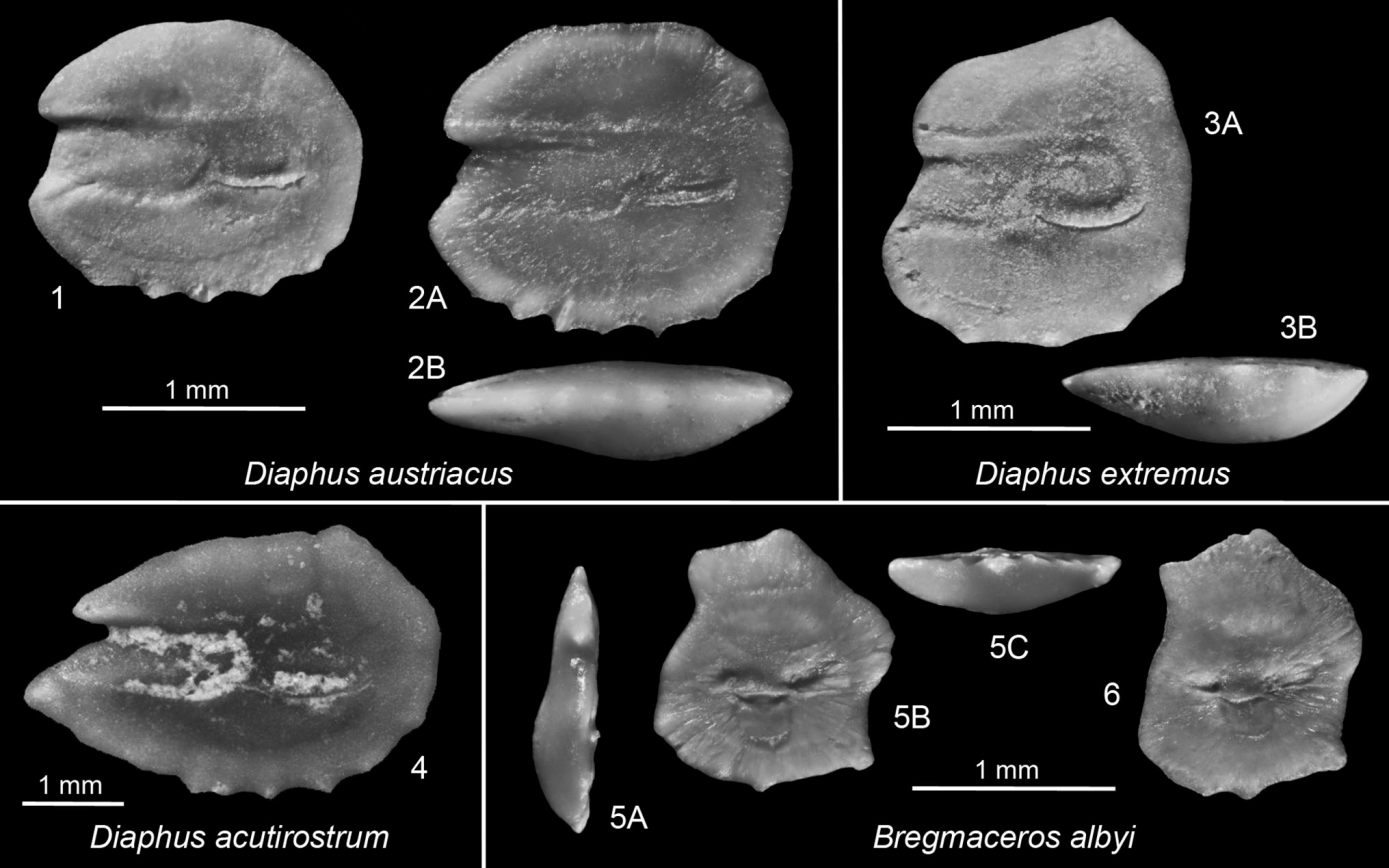
*1*–*2* *Diaphus austriacus* (Koken 1891). *1* Slanci-5, early Badenian; *2* Slanci-15, early Badenian, ventral view (*2B*). *3 Diaphus extremus* Schwarzhans 2010. Slanci-5, early Badenian, ventral view (*3B*). *4* *Diaphus acutirostrum* (Holec 1975). Slanci-15, early Badenian (otolith fragmented after photography). *5*–*6* *Bregmaceros albyi* (Sauvage 1880). *5* Slanci-16, early Badenian, anterior view (*5A*), ventral view (*5C*); *6* Slanci-13, early Badenian

*Material*: 1 specimen, Slanci, early Badenian, IGOTSL15/1.

*Diaphus austriacus* (Koken 1891) (Fig. 5.1, 2)

*Material*: 12 specimens, Slanci, early Badenian, IGOTSL15/2, 14/1-4, 5/1-3, 4/1-3, 3/1.

*Remark*. See Schwarzhans and Aguilera (2013) for extensive discussion about the validity of the species and extensive synonymy listing.

*Diaphus extremus* Schwarzhans 2010 (Fig. 5.3)

*Material:* 2 specimens, Slanci, early Badenian, IGOTSL7/1, 5/4.

*Remark*. *Diaphus extremus* is characterized by a very compressed shape with a ratio OL:OH of 0.85–0.9 (up to 0.95 in Slanci) and 4 denticles on the ventral rim. This is the first record of the species in the Paratethys. So far, it has been recorded only from time equivalent strata in the North Sea Basin.

*Diaphus kokeni* (Prochazka 1893)

*Material*: 3 specimens, Slanci, early Badenian, IGOTSL14/5, 7/2, 2/1.

Order Gadiformes Goodrich, 1909

Family Bregmacerotidae Gill, 1872

Genus *Bregmaceros* Thompson, 1840

**Fig. 6:**
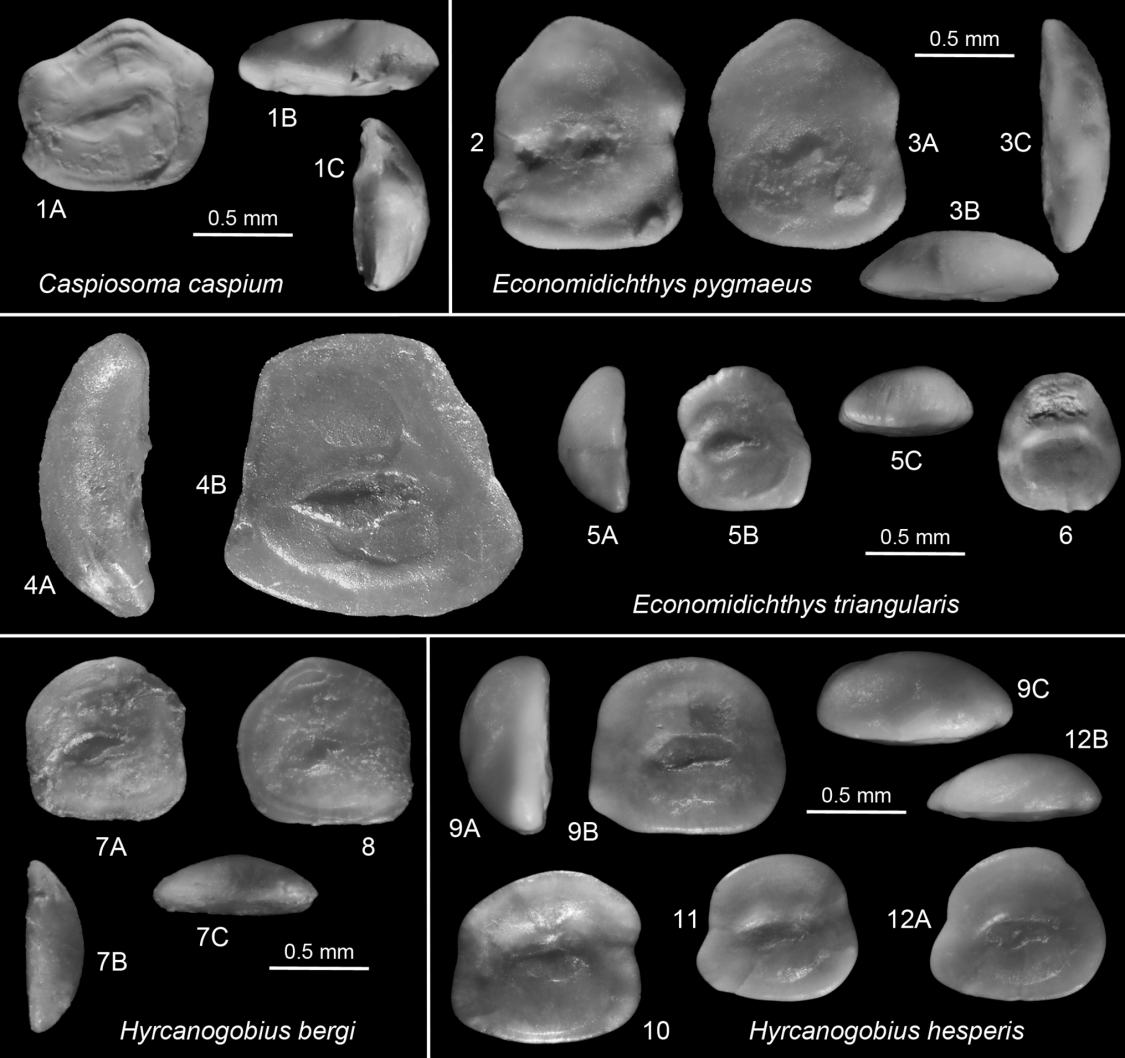
*1* *Caspiosoma caspium* (Kessler 1877). Recent, ZMMU P.13965 (male specimen), Ukraine, Black Sea, Lake Dniestrovski, Karagvolski Bay, SL = 28.5 mm, dorsal view (*1B*), posterior view (*1C*). *2*–*3* *Economidichthys pygmeus* (Holly 1929). Recent, BMNH 1999.4.23.231–290, Greece, Lake Trichonis, dorsal view (*3B*), posterior view (*3C*). *4*–*6* *Economidichthys triangularis* (Weiler 1943). *4* (mirrored): Holotype, SMF P 2651a, late Badenian (upper Buglovian), Salcia, Romania, anterior view (*4A*); *5*: IGOTBAB3/2, Barajevo-3, 40–43 m, Sarmatian, early Volhynian, anterior view (*5A*), dorsal view (*5*C); *6*: IGOTBAB3/3, Barajevo-3, 19–22 m, Sarmatian, late Volhynian. *7*–*8* *Hyrcanogobius bergi* Iljin, 1928. Recent, ZMMU P.4658, northern Caspian Sea, SL = 20–23 mm, posterior view (*7B*), dorsal view (*7C*). *9*–*12 Hyrcanogobius hesperis* n.sp. *9* Holotype, IGOTBAB4/1, Barajevo-4, 10–12 m, Sarmatian, late Volhynian, anterior view (*9A*), dorsal view (*9C*); *10* Paratype, IGOTBAB1/1, Barajevo1, 5560 m, latest Badenian; *11* Paratype, IGOTBAB1/3, Barajevo-1, 15–20 m, Sarmatian, middle Volhynian; *12* Paratype, IGOTBAB1/4, Barajevo-1, 20–25 m, Sarmatian, middle Volhynian, dorsal view (*12B*)

*Bregmaceros albyi* (Sauvage 1880) (Fig. 5.5, 6)

*Material:* 8 specimens, Slanci, early Badenian, IGOTSL18/1, 16/1, 15/3, 14/6, 13/1-3, 12/1.

Family Gadidae Rafinesque, 1810

Genus *Gadiculus* Guichenot, 1850

*Gadiculus argenteus* Guichenot, 1850

*Material:* 1 specimen, Slanci, early Badenian, IGOTSL13/4.

Genus *Palimphemus* Kner, 1862

*Palimphemus* cf. *minusculoides* (Schubert, 1912)

*Material:* 1 specimen, Barajevo B-3, 22–25 m, late Volhynian, IGOTBAB3/1.

*Remarks.* The single, small, juvenile specimen is somewhat eroded and, therefore, only tentatively placed in the species. See Bratishko et al. (2015) for extensive discussion about the validity of the species and extensive synonymy listing.

Order Perciformes Bleeker, 1859

Suborder Gobioidei Jordan and Evermann, 1896

Family Gobiidae Bonaparte, 1832

*Remark*. For comparison reasons, otoliths of the following small Recent Ponto-Caspian gobiid species are figured: *Caspiosoma caspium* (Kessler 1877) (Fig. 6.1), *Economidichthys pygmaeus* (Holly 1929) (Fig. 6.2, 3), *Hyrcanogobius bergi* Iljin, 1928 (Fig. 6.7, 8), *Knipowitschia caucasica* (Berg 1916) (Fig. 7.1, 2), *K. longecaudata* (Kessler 1877) (Fig. 7.6, 8), *K. panizzae* (Verga 1841) (Fig. 7.3, 4), *K. thessala* (Vinciguerra 1921) (Fig. 7.5), *Proterorhinus marmoratus* (Pallas 1814) (Fig. 8.6, 7), *P. nasalis* (De Filippi 1863) (Fig. 8.8) and *P. semilunaris* (Heckel 1837) (Fig. 8.9, 10).

**Fig. 7:**
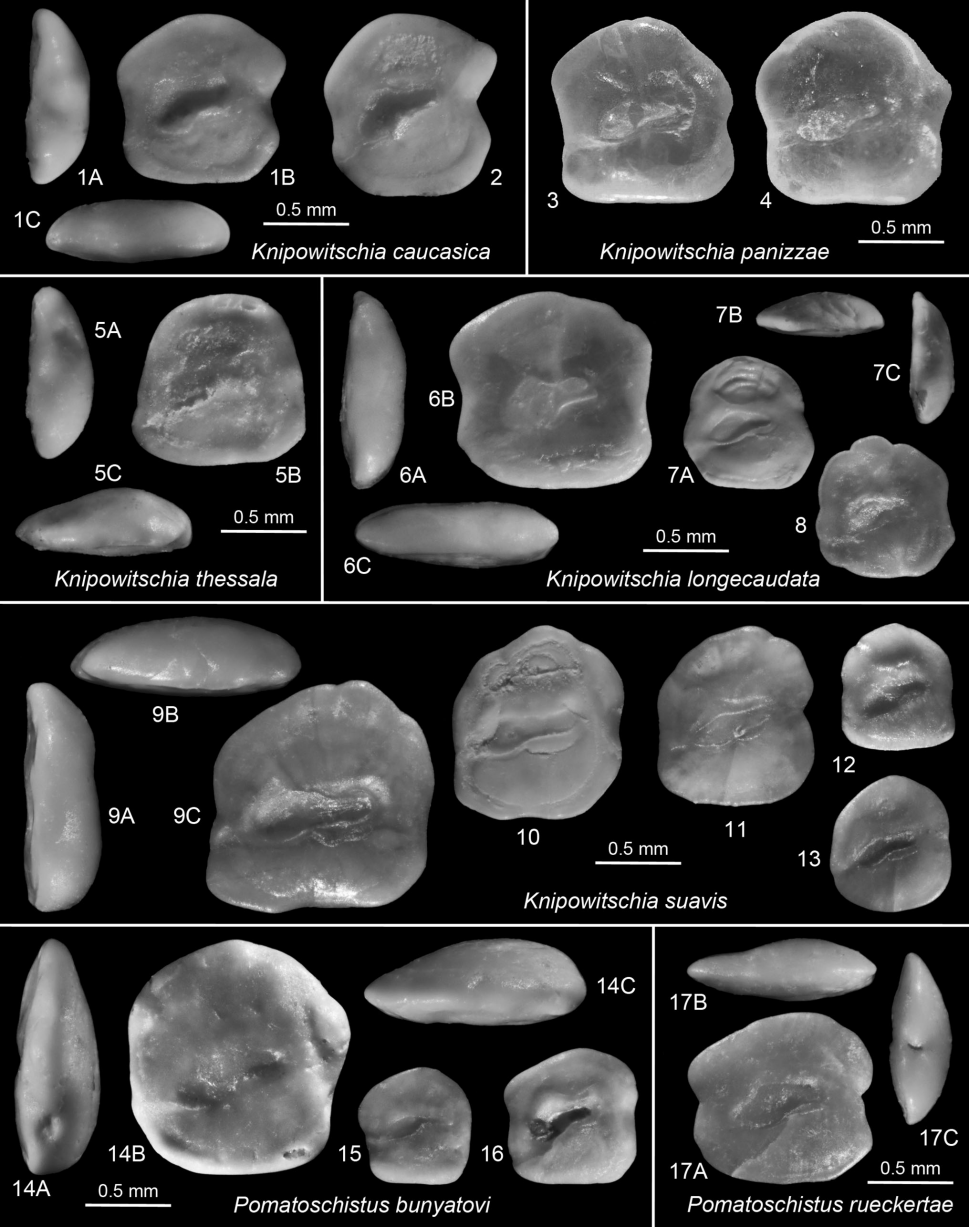
*1*–*2* *Knipowitschia caucasica* (Berg 1916). Recent, BMNH 1989.3.15.59-124, Greece, Lake Volvi, posterior view (*1A*), dorsal view (*1C*). *3*–*4* *Knipowitschia panizzae* (Verga 1841). Recent, Croatia, Zadar, TL = 39 mm, refigured (mirrored) from Lombarte et al. (2006). *5* *Knipowitschia thessala* (Vinciguerra 1921). Recent, BMNH 1989.3.15.33-58, Greece, Mati Tyrnavo, posterior view (*5A*), dorsal view (*5C*). *6*–*8* *Knipowitschia longecaudata* (Kessler 1877). Recent; *6*, *8* ZMMU P.22462, Ukraine, Black Sea, Lake Sassyk, 45°32′N, 29°39′E, SL = 28 (*6*), SL = 24 (*8*), posterior view (*6A*), dorsal view (*6C*); *7* ZMMU P.22452, Ukraine, Donau Delta, Lake Catlapug, 45°24′N, 29°02′E, SL = 22, dorsal view (*8B*), posterior view (*8C*). *9*–*13* *Knipowitschia suavis* Schwarzhans 2014. *9*, *12* (both mirrored): Turkey, Karaman Basin, Seythasan, Serravallian, posterior view (*9A*), dorsal view (*9C*); *10*, *13* (mirrored): IGOTBAB1/5, Barajevo 1, 55–60 m, latest Badenian; *11* (mirrored): IGOTBAB2/1, Barajevo-2, 57–62 m, Sarmatian, early Volhynian. *14*–*16* *Pomatoschistus bunyatovi* Bratishko, Schwarzhans and Reichenbacher, 2015. *14* (mirrored): IGOTBAB1/8, Barajevo-1, 85–87 m, late Badenian, posterior view (*1A*), dorsal view (*1C*); *15*–*16*: IGOTBAB1/9-10, Barajevo-1, 20–25 m, Sarmatian, Middle Volhynian. *17* (mirrored) *Pomatoschistus rueckertae* Schwarzhans, 2014. Turkey, Karaman Basin, Seythasan, Serravallian, dorsal view (*17B*), posterior view (*17C*)

**Fig. 8:**
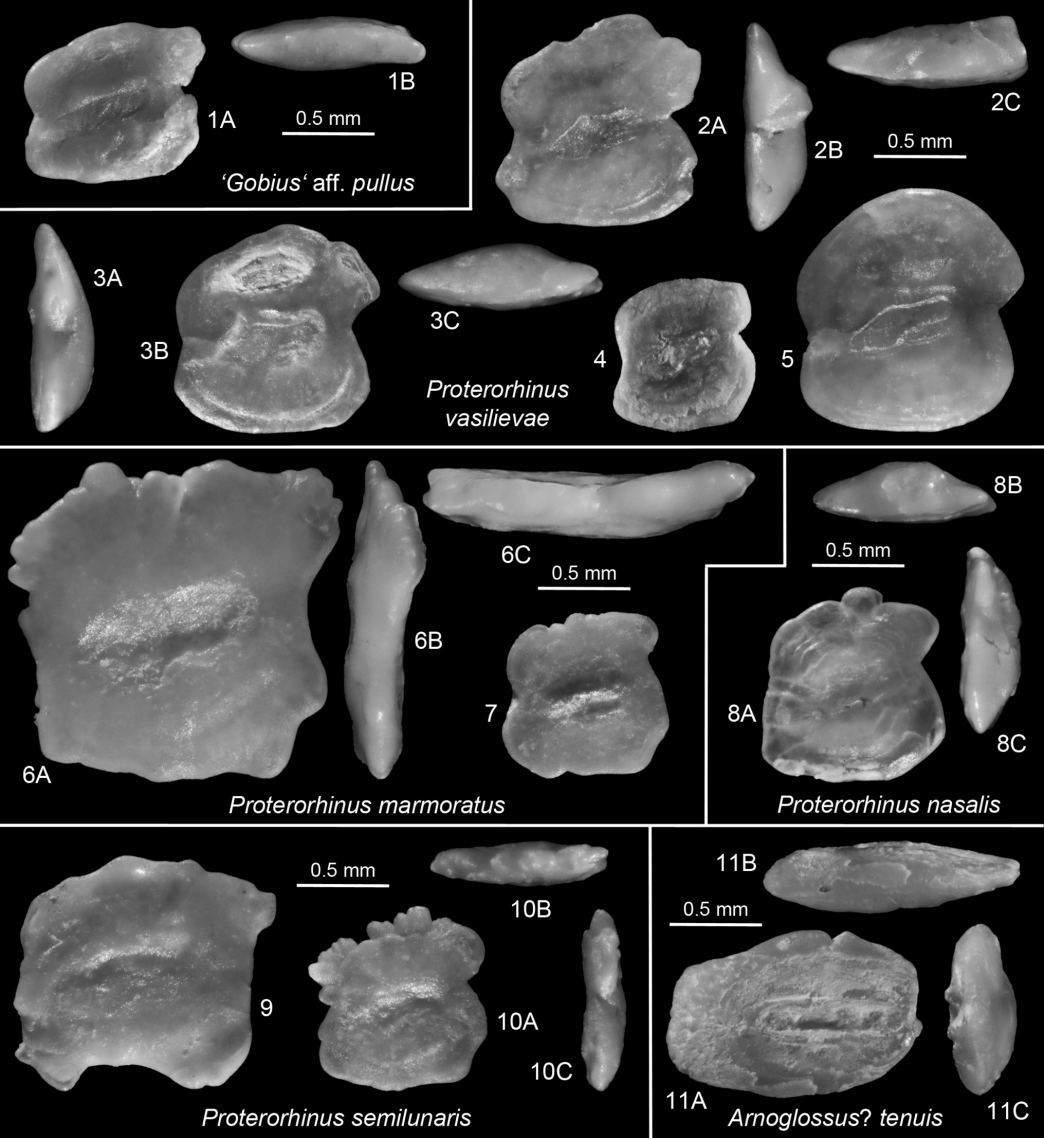
*1* “*Gobius”* aff*. pullus* Kramberger-Gorjanovic 1883. IGOTBAB1/11, Barajevo-1, 65–70 m, late Badenian, ventral view (*1*). *2*–*5* *Proterorhinus vasilievae* n.sp. *2* Holotype, IGOTBAB1/12, Barajevo-1, 60–65 m, late Badenian, posterior view (*2B*), dorsal view (*2C*); *3* (mirrored): Paratype, IGOTBAB1/13, Barajevo-1, 80–85 m, late Badenian, posterior view (*3A*), dorsal view (*3C*); *4* (mirrored): Paratype, IGOTBAB3/4, Barajevo-3, 61–64 m, Sarmatian, early Volhynian; *5* (mirrored): Paratype, IGOTBAB1/17, Barajevo-1, 60–65 m, late Badenian. *6*–*7* *Proterorhinus marmoratus* (Pallas 1814). Recent; *6* ZMMU P.21488, Aegean Sea, SL = 62 mm; *7*: ZMMU P.22465, Ukraine, Donau Delta, 45°20′N, 28°49′E, SL = 34 mm. *8*. *Proterorhinus nasalis* (De Filippi 1863). Recent, ZMMU P.22287, Russia, river Don near Woronesch, SL = 35 mm. *9*–*10* *Proterorhinus semilunaris* (Heckel 1837). Recent, ZMMU P.22779, Ukraine, Donau Delta, 45°22′N, 28°58′E; 9: SL = 47 mm; 10: SL = 43 mm, dorsal view (*10B*), posterior view (*10c*). *11* *Arnoglossus*? *tenuis* (Schubert 1906). IGOTBAB1/19, Barajevo-1, 25–30 m, Sarmatian, middle Volhynian, ventral view (*11B*), anterior view (*11C*)

Genus *Economidichthys* Bianco, Bullock, Miller and Roubal, 1987

Type species. *Gobius pygmaeus* Holly, 1929, type by original designation, Lefkas, Greece, freshwater; Recent.

*Economidichthys triangularis* (Weiler 1943)

Figure 6.4–6

?1906 *Otolithus* (*Gobius*) *intimus* Prochazka 1893; Schubert 1906: pl. 6, fig. 36 (non 35, 37)

1943 *Gobius triangularis*; Weiler 1943: pl. 1, figs. 25–26

1949 *Gobius triangularis* Weiler 1943, 1949: pl. 3, fig. 25, pl. 4, fig. 26

?1968 *Gobius intimus* Prochazka 1893; Rado 1968: pl. 4, fig. 3

?1968 *Otolithus* (*Gobius*) *sarmatus* Suzin 1968 (in Zhizhenko): pl. 17, fig. 2; (name not available: ICZN article 13.1.1)

1968 *Otolithus* (Gobius) *tenuis* Suzin 1968 (in Zhizhenko): pl. 17, fig. 5; (name not available: ICZN article 13.1.1)

1970 *Gobius triangularis* Weiler 1943; Stancu 1970: pl. 1, figs. 1–5

1974 *Gobius triangularis* Weiler 1943; Brzobohaty and Stancu 1974: pl. 2, figs. 1–10

1982 *Gobius triangularis* Weiler 1943; Strashimirov 1982: pl. 2, figs. 5–10, ?11–14

2006 *Gobius triangularis* Weiler 1943; Djafarova 2006: pl. 19, fig. 5, pl. 20, figs. 1–4

2010 *Trimma triangularis* (Weiler 1943); Schwarzhans 2010: pl. 104, fig. 6

*Material.* 2 specimens (IGOTBAB3/2-3), Barajevo B-3; 1 specimen 40-43 m, early Volhynian; 1 specimen 19–22 m, late Volhynian.

*Description.* Small, compressed otoliths up to about 1.8 mm length and with roughly triangular outline. OL:OH = 0.9–1.05; OH:OT = 2.2–2.6. Anterior and posterior rims inclined towards very short, flat, or rounded dorsal rim. Ventral rim flat or slightly curved, almost twice as long as dorsal rim. Anterior and posterior tips angular to rounded, low, close to ventral rim. No postdorsal or preventral projections; occasionally slight incision of anterior rim at about level of ostium.

Inner face flat. Sulcus small, short, centrally positioned, occasionally slightly anterior, somewhat deepened, slightly inclined. OL:SuL = 2.0–2.3. Sulcus inclination less than 10°.Ostium much wider and longer than cauda. Narrow long subcaudal iugum below entire cauda. Dorsal depression large, wide; ventral furrow distinct, wide, running far from ventral rim of otolith. Outer face distinctly convex, smooth.

*Ontogeny and Variability*. Brzobohaty and Stancu (1974) figured a complete ontogenetic sequence showing that smaller specimens expectedly are more rounded in outline than large ones. The here refigured holotype (Fig. 6.4) of about 1.6 mm length and a specimen figured by Brzobohaty of 1.8 mm length are the largest specimens known to date, and they both show the typical triangular outline. The two specimens from Barajevo also show some variation in the expression of the anterior rim (indented in the specimen of Fig. 6.5, smooth in 6.6) and position of the sulcus (slightly forward positioned in Fig. 6.5, centrally positioned in Fig. 6.6).

*Discussion.* This small and characteristic triangular shaped otolith has been widely reported from the late Badenian and the Sarmatian of the Central and Eastern Paratethys. Schwarzhans (2010) related it to the dwarf reef goby genus *Trimma* because of its small sulcus and the triangular shape, but otoliths of the Ponto-Caspian genus *Economidichthys* were not then known. Now otoliths of *E. pygmaeus* (Fig. 6.2, 3) reveal even better correlation, as do the otoliths of *Knipowitschia thessala* (Fig. 7.5), a representative of one of three species groups within the genus *Knipowitschia* which shares with *Economidichthys* the reduced squamation and its adaption to freshwater habitats in the Adriatic and Aegean drainage systems (Miller 2004). He summarized earlier investigations by concluding that the origin of both groups may be linked to the early Pliocene ‘Lago Mare’ development. The widespread occurrence of *E. triangularis* in marginal marine to brackish environment, if taken at face value, indicates that their origin could indeed be somewhat earlier and would find its roots in the segregated development of the Sarmatian seas of the Paratethys. Molecular studies of the extant species of the genera involved might be able to shed more light into timing and phasing of the evolution of this complex group, when they become available.

*Economidichthys triangularis* has been recorded from the late Badenian and Sarmatian s.s. of the Transylvanian Basin, the Dacian Basin, the northern Caucasus and Azerbaijan and its distribution is now extended into Serbia. Brzobohaty and Stancu (1974) mentioned occurrence also in the Pannonian and Slovakian basins.

A single earlier reference from the early Badenian of Austria in Schubert (1906; Vöslau, Upper Lagenidae Zone) and referenced by Brzobohaty and Stancu (1974) requires further verification. There are multiple records of similar-looking small gobiid otoliths from early Badenian sediments, which, however, appear to be generally more rounded and less high and triangular in outline. Nominal species of such appearance are *Gobius praeclarus* Prochazka 1893, *Gobius rotundus* Pobedina 1954, *Gobius rotundus tarchanensis* Pobedina 1954, and *Gobius rotundus tshokrakrensis* Strashimirov 1980. The nature of all these nominal species cannot be reliably assessed without review of the type-material because of their less than optimal documentation, and since the specimens appear to be difficult to track, one has to perceive those names as nomina dubia for the time being.

Genus *Gobius* Linnaeus, 1758

Type species. *Gobius niger* Linnaeus, 1758, type by subsequent designation, Europe; Recent.

‘*Gobius*’ aff. *pullus* Kramberger-Gorjanovic 1883

Figure 8.1

?1883 *Gobius pullus*; Kramberger-Gorjanovic 1883: pl. 25, figs. 2, 2a

*Material.* 2 specimens (IGOTBAB1/11, 4/6); 1 specimen Barajevo B-1, 65–70 m, late Badenian; 1 specimen Barajevo B-4, 12–14 m, late Volhynian.

*Description.* Moderately elongate, thin otolith. OL:OH = 1.15; OL:OT = 3.0. Outline parallelogramlike, with rather short preventral and postdorsal projections; predorsal and postventral angles orthogonal. Ventral rim flat, horizontal; dorsal rim with broadly rounded mediodorsal angle; anterior and posterior rims with slight indentation at level of sulcus.

Inner face moderately convex, postdorsal projection moderately bent outwards. Sulcus slightly supramedian, narrow, its inclination about 20°; no ostial lobe; faint and very narrow subcaudal iugum. Dorsal depression indistinct; ventral furrow broad, but with indistinct margin. Outer face slightly concave.

*Discussion*. One of us (Schwarzhans) recently received a photograph of one of the two syntypes of *G. pullus* from the Sarmatian of Croatia containing an otolith in situ, which appears to be very similar to the two specimens described here from the late Badenian and Sarmatian of Serbia. The final correlation will be subject to re-study of the original specimens of Kramberger-Gorjanovic (1883). Also, the taxonomic position in the genus *Gobius* must be seen as preliminary. Kramberger-Gorjanovic noted 28 vertebrae, I + 8 rays in the second dorsal fin and I + 9 rays in the anal fin. While the number of vertebrae agrees with the genus *Gobius* (and many other extant genera, but not the Ponto-Caspian endemics *Neogobius* or *Ponticola*), the very low numbers of rays in the second dorsal and the anal fins are not matched. We, therefore, expect that a review of the skeletons will point to another generic allocation of the species.

The otoliths described here closely resemble *Gobius dorsorostralis* Weinfurter, 1954, which has been recorded from brackish water environments of the Sarmatian to Pannonian of Austria and of the Serravallian of SE Turkey (Schwarzhans 2014). The identity of both nominal species is subject to review of the specimens of *G. pullus* with otolith in situ. Also similar to some extent are *Neogobius udovichenkoi* and *Ponticola zosimovichi*, recently described by Bratishko et al. (2015) from he Konkian (Late Badenian equivalent of the Eastern Paratethys) of Kazakhstan, but are more compressed than both of them and differ further in the very narrow ostium without ostial lobe.

Genus *Hyrcanogobius* Iljin, 1928

Type species. *Hyrcanogobius bergi* Ijin, 1928, type by monotypy, Caspian Sea off the Volga, Ural, and Emba estuaries; Recent.

*Hyrcanogobius hesperis* n.sp.

Figures 6.9–12

*Etymology.* From Latin *hesperis*, westerly, referring to the western occurrence of the species when compared to the Recent *H. bergi*.

*Type material*. Holotype: IGOTBAB4/1 (Fig. 6.9). Paratypes: 8 specimens; Barajevo B-1, 55–60 m, late Badenian (2 specimens, IGOTBAB1/1-2); Barajevo B-1, 20–25 m, middle Volhynian (IGOTBAB1/3); Barajevo B-1, 15–20 m, middle Volhynian (IGITBAB1/4); Barajevo B-4, 32–34 m, early Volhynian (IGOTBAB4/2); Barajevo B-4, 28–30 m, early Volhynian (IGOTBAB4/3); Barajevo B-4, 20–22 m, middle Volhynian (IGOTBAB4/4); Barajevo B-4, 18–20 m, late Volhynian (IGOTBAB4/5).

*Type locality*. Barajevo B-4 well, 10–12 m; south of Belgrade, Serbia.

*Age*. Late Volhynian, late Sarmatian, late Serravallian, middle Miocene.

*Diagnosis*. Roundish, compact otoliths; OL:OH = 1.05–1.15. All rims smoothly rounded with broadly rounded pre- and postdorsal and postventral angles; preventral angle sometimes slightly projecting. Inner face flat; outer face strongly convex. Sulcus very small, deepened, very little inclined; OL:SuL = 2.2–2.5; ostium not much wider than cauda; no or very thin caudal iugum.

*Description* (*n* = 5). Compressed, thick otoliths reaching slightly more than 1 mm of length; OL:OT = 2.0–2.3. Anterior and posterior rims sub-vertical, slightly inclined towards rounded dorsal rim. Pre- and postdorsal angles gently rounded, no postdorsal projection. Ventral rim flat to very slightly curved, somewhat longer than dorsal rim. Preventral angle slightly and variably projecting rostrumlike, but always well rounded; postventral angle well rounded. All rims smooth. No incisions on anterior rim, rarely weak incision on posterior rim.

Inner face flat to slightly convex. Sulcus very small, short, narrow, centrally positioned, distinctly deepened, only very slightly inclined. Sulcus inclination less than 10°. Ostium only slightly wider than cauda. No or narrow long subcaudal iugum below entire cauda. Dorsal depression moderately large, indistinct; ventral furrow wide, moderately distinct, running far from ventral rim of otolith. Outer face distinctly convex, smooth.

*Comparison*. Despite an unspectacular appearance, otoliths of *H. hesperis* are easily recognized by the small, narrow sulcus, the rounded rectangular outline with a slightly pronounced preventral angle and the very narrow or lacking caudal iugum. Many of the small gobiid otoliths described from Sarmatian strata in the past are difficult to assess without review of the original material, but there appears to be none that would share all pertinent diagnostic features with *H. hesperis*.

*Hyrcanogobius hesperis* resembles well the otoliths of the recent endemic Ponto-Caspian *H. bergi*, the only extant species of the genus, in general appearance, outline, and the size, depth, and format of the sulcus, but differs in being slightly more elongate (OL:OH = 1.05–1.15 vs 0.95–1.05), showing more strongly pronounced preventral angle and a very narrow or no caudal iugum (vs. caudal iugum much expanded on the expanse of the width of the cauda). We interpret *H. hesperis* as an early representative of this endemic genus close to its basal dichotomy supposedly from the *Knipowitschia* group (Miller 2004).

Genus *Knipowitschia* Iljin, 1927

Type species. *Gobius longecaudatus* Kessler, 1877, type by monotypy, southern and middle Caspian Sea; Recent.

*Knipowitschia suavis* Schwarzhans 2014

Figures 7.9–13

2014 *Knipowitschia suavis*; Schwarzhans, 2014: pl. 10, figs. 7–14.

2015 *Knipowitschia suavis* Schwarzhans, 2014; Bratishko, Schwarzhans, Reichenbacher, Vernyhorova and Ćorić, 2015: figs. 10.7–10.12

*Material.* 4 specimens (IGOTBAB1/5-7, 2/1); 3 specimens Barajevo B-1, 55–60 m, late Badenian; 1 specimen Barajevo B-2, 57–62 m, early Volhynian.

*Discussion*. This species is best recognized by the dorsal rim with its mediodorsal angle and the lack of a postdorsal projection, and the very long subcaudal iugum (for specific gobiid otolith terminology see Schwarzhans 2014). The few specimens from the Barajevo wells are relatively small, but exhibit these characters well enough for a specific identification. They represent the first record of the species in the Central Paratethys. Other records are from the Serravallian of the northern Caspian Basin (Mangyshlak) (Bratishko et al. 2015) the Karaman Basin, SE Turkey (Schwarzhans 2014). Two otoliths from the Karaman Basin are figured for comparison (Fig. 7.9, 10). With these new records, *K. suavis* is one of the geographically most widespread gobiid species in the middle Miocene of the Paratethys and eastern Mediterranean.

The genus *Knipowitschia* comprises 17 valid Recent species (Froese and Pauly 2014), many of which are freshwater and endemic to relatively limited areas in eastern Europe. Otoliths are known from five of those and are figured herein from four—*K. caucasica* (Fig. 7.1–2), *K. panizzae* (Fig. 7.3–4), *K. longecaudata* (Figs. 7.6–8) and *K. thessala* (Fig. 7.5). Miller (2004) recognized three species groups of which the first two belong to the *K. caucasica* species group and the latter two to the *K. longecaudata* and *K. punctatissima* groups respectively. *Knipowitschia suavis* resembles most *K. panizzae* and we therefore suppose it being related to the *K. caucasica* group.

Genus *Pomatoschistus* Gill, 1864

Type species. *Gobius minutus* Pallas, 1770, type by original designation, Belgian Sea; Recent.

*Pomatoschistus bunyatovi* Bratishko, Schwarzhans and Reichenbacher, 2015

Figure 7.14–16

1966 *Gobius praeclarus* Prochazka 1893; Smigielska 1966: pl. 19, figs. 2, 3

1969 *Gobius vicinalis* Koken 1891; Stancu 1970: pl. 4, fig. 2 (non 1, 3)

1974 *Gobius telleri* Schubert 1906; Brzobohaty and Stancu, 1974: pl. 1, fig. 2

1992 “genus Gobiidarum” sp. 1; Radwanska 1992: pl. 35, figs. 1, 2, text-fig. 146

1992 “genus Gobiidarum” sp. 5; Radwanska, 1992: pl. 35, figs. 3, 4, text-fig. 150

2006 *Pomatoschistus laevis* Weiler 1942; Djafarova 2006: pl. 19, figs. 1–3 (non pl. 18, fig. 4)

2015 *Pomatoschistus bunyatovi*; Bratishko, Schwarzhans, Reichenbacher, Vernyhorova and Ćorić, 2015: figs. 10.13–10.17

*Material.* 3 specimens (IGOTBAB1/8-10); 1 specimen Barajevo B-1, 85-87 m, late Badenian; 2 specimens Barajevo B-1, 20–25 m, middle Volhynian.

*Description.* Small, thick otoliths with regular, rounded to rectangular outline, dorsally wider than ventrally and up to about 1 mm length. OL:OH = 0.95–1.05; OH:OT = 2.7–3.2. Dorsal rim gently curving without prominent angles, highest at about middle. Ventral rim shallow. Anterior and posterior rims inclined resulting in longest axis of otolith located distinctly above centre, mostly above sulcus.

Inner face flat to slightly convex and smooth. Sulcus narrow, deepened, inclined at about 15°. Ostial lobe very low; no or very faint and thin subcaudal iugum. Dorsal depression shallow; ventral furrow running at some distance from ventral rim. Outer face convex, smooth.

*Discussion.* *Pomatoschistus bunyatovi* is a typical species of the genus *Pomatoschistus* characterized by the regular, high outline and the narrow sulcus with the low ostial lobe. *P. bunyatovi* is a geographically rather widely distributed gobiid that seems to occur throughout the entire Paratethys during the Serravallian. In the Serravallian of the Karaman Basin of the eastern Mediterranean it is replaced by the persistent *P. quagga* (Heckel 1837) and *P. rueckertae* Schwarzhans 2014. The former differs in being even more high-bodied (OL:OH = 0.9–0.95 vs. 0.95–1.05), while *P. rueckertae* (Fig. 7.17) is more elongate (OL:OH = 1.05–1.15), and both species show a more convex and smoother inner face. Pomatoschistus otoliths usually do not show a subcaudal iugum, but in rare instances a thin and indistinct iugum may be present, such as in certain specimens figured here of *P. bunyatovi* (Fig. 7.16) and *P.* *rueckertae* (Fig. 7.17).

Genus *Proterorhinus* Smitt, 1899

Type species. *Gobius marmoratus* Pallas, 1814, type by original designation, Sevastopol, Ukraine; Recent.

*Proterorhinus vasilievae* n.sp.

Figure 8.2–5

*Etymology.* Named in honour of Katerina Vasilieva (ZMMU, Moscow), in recognition of her many contributions to the knowledge of Recent Ponto-Caspian gobies and her support in achieving an otolith database of these fishes of the senior author.

*Type material.* Holotype: IGOTBAB1/12 (Fig. 8.2). Paratypes: 7 specimens; Barajevo B-1, 80–85 m, late Badenian (IGOTBAB1/13); Barajevo B-1, 75–80 m, late Badenian (2 specimens IGITBAB1/1415); Barajevo B-1, 65–70 m, late Badenian (IGOTBAB1/16); Barajevo B-1, 60–65 m, late Badenian (IGOTBAB1/17); Barajevo B-1, 55–60 m, late Badenian (IGOTBAB1/18); Barajevo B-3, 61–64 m, early Volhynian (IGOTBAB3/4).

*Type locality.* Barajevo B-1 well, 60–65 m; south of Belgrade, Serbia.

*Age.* Late Badenian, early Serravallian, middle Miocene.

*Diagnosis.* Compressed otoliths; OL:OH = 0.9–0.95; OH:OT = 3.0–3.3. Dorsal rim highest at postdorsal angle; predorsal angle low, distinct, rounded; postdorsal projection short, positioned high. Inner face slightly convex. Sulcus narrow, inclined at 15–20°; caudal iugum moderately wide and long, extending below cauda to about 2/3 from indentation at ostial/caudal joint.

*Description* (*n* = 5). Compressed, relatively thin otoliths reaching to about 1.3 mm of length. Dorsal rim anteriorly depressed with distinct, but broadly rounded predorsal angle; highest point at about 2/3 from anterior tip at broad postdorsal angle; postdorsal projection broad, slightly projecting, positioned high on posterior rim. Dorsal rim irregularly undulating to almost smooth. Anterior and posterior rims nearly vertical; anterior rim with broad, shallow concavity at level of ostium; posterior rim with sharp indention below postdorsal projection at about level of tip of cauda. Ventral rim shallow, very slightly curved, smooth. Preventral angle distinct, not projecting; postventral angle broadly rounded. Rims mostly thin.

Inner face slightly bent along horizontal axis. Sulcus small, short, narrow, centrally positioned, distinctly inclined at about 15–20°. OL:SuL = 2.0; ostium wider than cauda, with marked ostial lobe. Moderately wide and long subcaudal iugum terminating slightly before end of cauda. Dorsal depression moderately large, mostly distinct, particularly ventrally; ventral furrow distinct, narrow, running relatively close to ventral rim of otolith. Outer face moderately convex, smooth, or with some ornamentation on dorsal field.

*Comparison.* These otoliths resemble those of the recent species of *Proterorhinus* in the sulcus proportions, orientation, and the presence of a rather long subcaudal iugum as well as in the otolith proportions, the massive, but short postdorsal projection and the often irregularly ornamented dorsal rim. The otoliths of the Recent species are mostly thinner and with a much less pronounced postdorsal angle, with those of *P. nasalis* being the thickest and thus resembling the fossil ones closest. Miller (2004) recognized a single species, but Froese and Pauly (2014) maintain a total of five species, with one based on a single holotype and another restricted to a very specific freshwater environment on the Crimea. The remaining three species are more widespread with *P. marmoratus* found in marine to brackish water environments and *P. nasalis* and *P. semilunaris* in freshwater to brackish water environments.

*Proterorhinus vasilievae* also resembles otoliths of the common early to middle Miocene, fully marine and widely distributed *Lesueurigobius vicinalis* (Koken 1891). Otoliths of *L. vicinalis* grow to much larger sizes (about 4 mm length) and small, juvenile specimens at about 1 mm length show a generalized, rounded outline. *Lesueurigobius vicinalis* differs from *P. vasilievae* in the more strongly inclined, straight predorsal rim, the higher dorsal rim (except fig. 8.5), the less incised anterior rim, the wider sulcus and wider, but shorter subcaudal iugum, all rather subtle differences each, but in combination resulting in a good distinction. There have been a number of superficially similar looking otoliths described from Sarmatian locations in previous publications that are not optimally documented and can only be reasonably assessed by reviewing of the original material, most of which unfortunately must be considered lost as to the current status of our investigations. These are: (1) *Hymenocephalus quadratus* Strashimirov, 1981 from the Karaganian of Bulgaria, which, judging from the iconography, differs in the regular oval sulcus, the postdorsal projection being shorter than the broad postventral angle and the shape of the dorsal rim. (2) A specimen identified as *Gobius vicinalis* Koken 1891 in Weiler (1949; pl. 4, fig. 29) from the late Badenian of Romania, which is characterized by a sharp, though not much projecting preventral angle. (3) Otoliths identified as *Gobius francofurtanus* Koken 1891 by Djafarova (2006) from the Sarmatian of Azerbaijan, which show a more projecting postdorsal process. (4) An otolith identified as *Gobius vicinalis* in Suzin (1968) from the Sarmatian of the northern Caucasus resembling very much in all aspects the paratype of Fig. 8.5.

Order Pleuronectiformes Bleeker, 1859

Suborder Pleuronectoidei Bleeker, 1859

Family Bothidae Regan, 1910

Genus *Arnoglossus* Bleeker, 1862

Type species. *Pleuronectes arnoglossus* Bloch and Schneider, 1801 (synonym of *Pleuronectes laterna* Walbaum, 1792), type by monotypy, no locality given; Recent.

*Arnoglossus*? *tenuis* (Schubert 1906)

Figure 8.11

*Material*. 1 specimen (IGOTBAB1/19) Barajevo B-1, 25–30 m, middle Volhynian.

*Discussion*. A single left otolith representing this somewhat enigmatic otolith-based flatfish species (see Bratishko et al. 2015, for extensive discussion and validation of the species). It differs slightly from the specimens recorded by Bratishko et al. in the narrower sulcus and the slightly expanded anterior-dorsal rim.

## Results and discussion

### The Badenian faunal change and the origin of the Ponto-Caspian gobies

The otoliths obtained from Slanci and the Barajevo wells represent a relatively small number of specimens and species. Nevertheless, their analysis reflects two very distinct effects, similar to observations recently discussed by Bratishko et al. (2015).

Mesopelagic fishes, primarily from the families Myctophidae and Bregmacerotidae, dominate the early Badenian otolith association. Following a stratigraphic gap of probably relatively short duration, otoliths of shallow water, near shore, to brackish marine fishes dominate the late Badenian and Sarmatian otolith associations. This drastic shift in the fish fauna reflects the massive environmental changes that the Paratethys experienced during this time interval (Harzhauser et al. 2002, 2003; Ilyina 2006; Kowalke and Harzhauser 2004; Lukeneder et al. 2011; Rögl 1998, 1999).

The other major effect observed in the otolith assemblages of the late Badenian and the Sarmatian is the rise of the Gobiidae, particularly of those with Ponto-Caspian affinities and Ponto-Caspian endemics. In fact, most major Ponto-Caspian gobiid genera have now been identified by otoliths from this time interval: *Economidichthys, Hyrcanogobius, Knipowitschia, Neogobius, Ponticola* (Bratishko et al. 2015), and *Proterorhinus*. The first three genera and *Pomatoschistus* represent the so-called sand gobies (Thacker and Roje 2011). *Economidichthys* in the Recent is restricted to the Dinarid freshwater system and is lacking from the Ponto-Caspian Basin proper. Economidis and Miller (1990) and Miller (1990) associated the dichotomy of the genera *Economidichthys* and *Knipowitschia* with the Lago Mare event of the Mediterranean during the early Pliocene, while they assumed the splitting of the precursors of both genera from *Pomatoschistus* to having potentially occurred “in the middle Miocene Serravallian (12–14 Ma), with enclosure of the brackish Sarmatic Sea and the beginning of the evolution of the Ponto-Caspian endemic fauna”. Huyse et al. (2004) were the first to undertake a molecular genome-based phylogeny of the “sand-gobies” and compared their evolution with the paleoclimatic history of the Mediterranean Basin. They too concluded a young origin of most of the genera and species involved, linking it to the Messinian crisis and younger events. Our data, in contrast, strongly suggest that the origin of the involved lineages may be older than assumed in these studies and were all related to the segregation of the Paratethys during the middle Miocene. In our interpretation of the systematic position of *E. triangularis* the genus *Economidichthys* had a Paratethyan origin and only subsequently would have become adapted to freshwater regimes, where it has survived until today. Similar brackish to freshwater adaptions in the Dinarids and the Ponto-Caspian Basin are observed in the related genus *Knipowitschia.* Still another related genus, *Hyrcanogobius*, is known in the Recent from a single endemic Ponto-Caspian species, but now is also indicated from the middle Miocene of the Paratethys. The large group of Ponto-Caspian gobies (Thacker and Roje 2011; Medvedev et al. 2013) is likewise represented since late Badenian in the Paratethys by the genera *Neogobius, Ponticola* (Bratishko et al. 2015) and now also *Proterorhinus.*

None of these gobiid genera have yet been identified prior to late Badenian times, and, except for *Knipowitschia suavis*, which was first described from the Serravallian of SE Turkey, none of them has been identified from locations outside of the Paratethys. The only widespread “sand goby” genus apparently was (and still is) *Pomatoschistus*, which is also recorded from the Mediterranean and the North Sea Basin and includes earlier records than of late Badenian age.

**Fig. 9:**
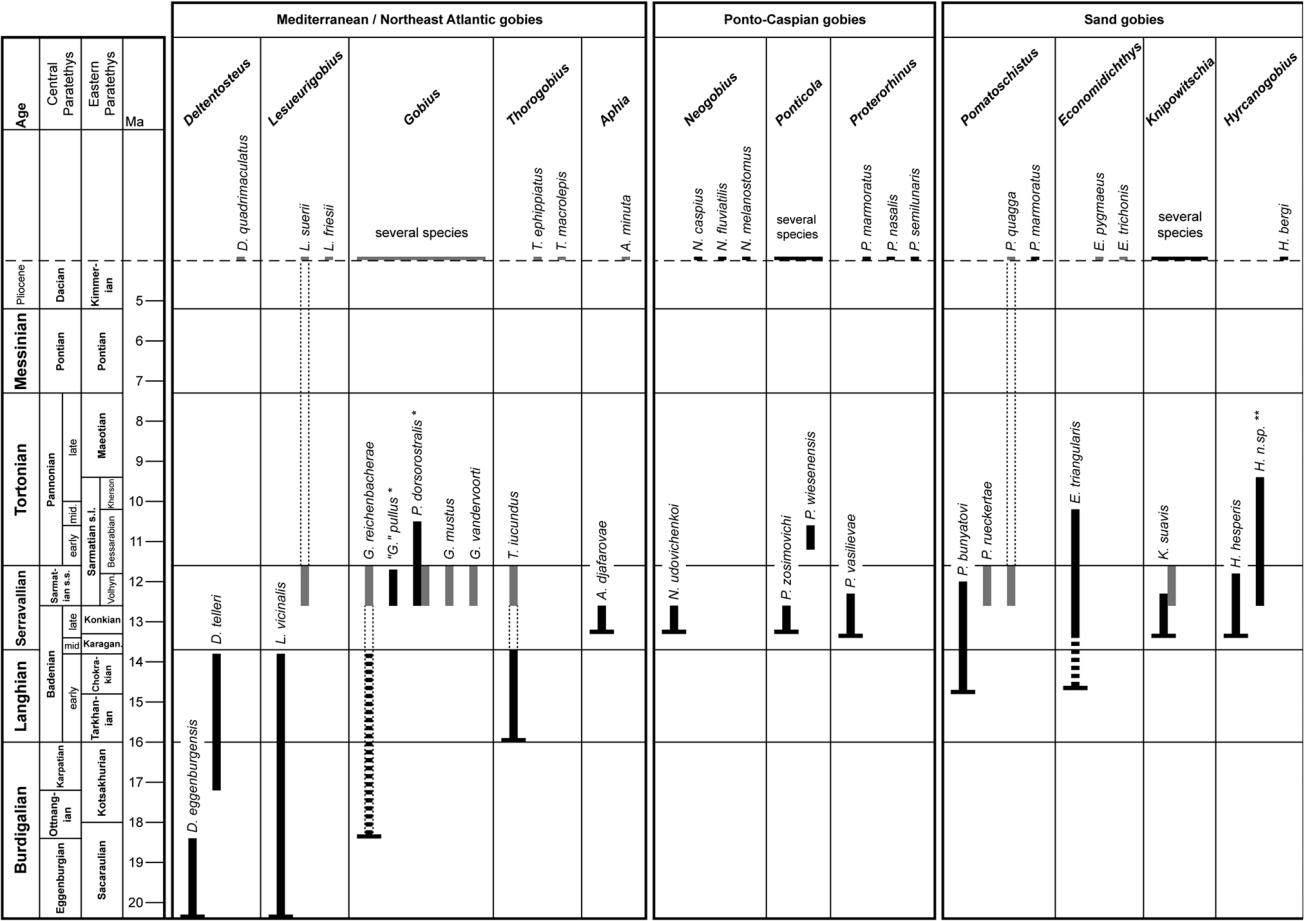
First occurrences of otolith-based gobiid species in the Miocene of the Paratethys and their link to the Recent Ponto-Caspian gobies. Stratigraphy after Gradstein et al. (2004); Central Paratethys stages after Piller and Erhart (2004), Pillar et al. (2007), Jimenez-Moreno et al. (2006), Pezelj et al. (2013) and Lukeneder et al. (2011); Eastern Paratethys stages after Nevesskaya et al. (2003) and Semenenko et al. (2011). Otolith data compiled from Brzobohaty et al. (2007), Schwarzhans (2010, 2014), Nolf (2013) and Bratishko et al. (2015); *black bars* Paratethys, *gray bars* Mediterranean. *Single* *asterisk* changed or problematic taxonomic position; *double asterisk* undescribed species from Bulgaria

Figure 9 summarizes the current knowledge of the stratigraphic occurrence of otolith-based Ponto-Caspian, Atlanto-Mediterranean and sand gobies in the Miocene of the Paratethys and the Mediterranean. Gobiid otoliths are readily recognized by their peculiar shoe-sole shaped sulcus and the general appearance. Species are mostly well defined, but one should be reminded that the allocation to specific genera in gobiid otolith morphologies often is somewhat ambiguous. Therefore, the interpretation of the allocation of the otolith-based species as presented above would be served well with some calibration with in situ otolith findings. So far, only a single Paratethyan skeleton-based goby has been identified with otoliths in situ, *Gobius pullus* Kramberger-Gorjanovic 1883, which, subject to outstanding review, does not seem to represent either a species of the genus *Gobius* nor any of the otolith-based species with Ponto-Caspian affinities.

### Paleobiogeographic distribution of gobiids in the Paratethys

A variety of hypotheses have been published in recent literature dealing with the paleogeography of the Paratethys during middle and late Miocene and particularly concerning the connectivity of the Paratethys to adjacent oceans as well as the interlinking of the Central and Eastern Paratethys basins. In the sequence of paleogeographic reconstructions of what is considered the major textbook in this respect by Rögl (1998), the Paratethys is shown to be interconnected to the Mediterranean in the NW as well as the SE, and also to the Persian Gulf in the SE during early Badenian. During middle Badenian, the Eastern Paratethys became entirely separated, while the Central Paratethys remained connected to the Mediterranean in the NW, but with parts of it having become segregated and subject to evaporation (Pre-Carpathian Trough and Transylvanian Basin). During late Badenian the NW connection became permanently aborted, while both Central and Eastern Paratethys were reconnected again. In the SE, Rögl shows a connection of the Eastern Paratethys with the Indian Ocean through the Persian Gulf, and a possible second interconnection to the Mediterranean. During the Sarmatian, the Eastern Paratethys is shown as still interconnected to the Central Paratethys and the Mediterranean in the SE, while the connection to the Persian Gulf became permanently aborted. Bartol et al. (2014) have found arguments for a much broader interconnectivity of the Paratethys during late Badenian including a persistent connection in the NW to the Mediterranean and more than one connection of the Mediterranean to the Eastern Paratethys.

We believe that fishes are particularly well suited for the study of marine connections due to their fast response time, for instance, as is evidenced by the so-called Lessepsian migration after the creation of the Suez Canal (Dov Por 1978). While fossil fish data from the Miocene of the Paratethys is still rather limited both from skeletons (Bannikov 2010; Carnevale et al. 2006) and otoliths, certain tendencies already emerge from those available that allow a careful reflection of the status. Certain groups appear to be particularly valuable in this respect: Clupeidae (mainly based on skeleton finds), Gadidae (both skeleton and otolith finds) and Gobiidae (primarily otolith finds). We will focus on the Gobiidae in the following because of the richness of Ponto-Caspian endemics in the Recent. Our knowledge about the spatial distribution of otolith-based gobiid species in the Middle and Late Miocene of the Paratethys is rapidly improving, but with the caveat that a substantial review of the earlier works of Pobedina (1954), Pobedina et al. (1956), Strashimirov (1972, 1980, 1981a, b, 1982, 1984, 1985a, b), and Djafarova (2006) is required (see also discussion in Bratishko et al. 2015).

The early Badenian indeed shows a rather uniform open marine fish fauna throughout the Paratethys and the Tethys (Bratishko et al. 2015) as would be expected. This phase is followed by a dramatic faunal break between early and late Badenian in the Central Paratethys and a likewise break in the Eastern Paratetheys between Chokrakian and Karaganian/Konkian (Bratishko et al. 2015). The open marine elements disappear, and the endemic evolution of fishes blossoms within these three families. This is also the time when many of the endemic Ponto-Caspian gobiid genera are first observed, as stated above, going hand-in-hand with fundamental shifts of the environments in the Paratethys (Kovač et al. 2007) that apparently favored gobiid evolution and diversification. A substantial otolith assemblage recently described from the Serravallian (Sarmatian equivalent) of the Karaman Basin in SE Turkey (Schwarzhans 2014) revealed a rich shallow marine fish fauna with many gobiid species and close afinities to the older fish fauna of the Central Paratethys, but very little resemblance indeed to any of the time equivalent fish faunas from the Paratethys. This observation is again confirmed by the late Badenian and Sarmatian otoliths now studied from Serbia. Therefore, we conclude that the fish fauna does not show support for a connection of the Paratethys with the Mediterranean during Serravallian times, neither in the NW as postulated by Bartol et al. (2014) nor in the SE, where the Karaman Basin would have been located ideally within the fairway of such putative waterway. A single species, the gobiid *Knipowitschia suavis*, is shared between the Mediterranean and the Paratethys during the Serravallian time. It appears to have been a species tolerant to a wide spectrum of salinities since it has been found in fully marine as well as brackish water facies, which may explain its wide geographic distribution.

**Fig. 10:**
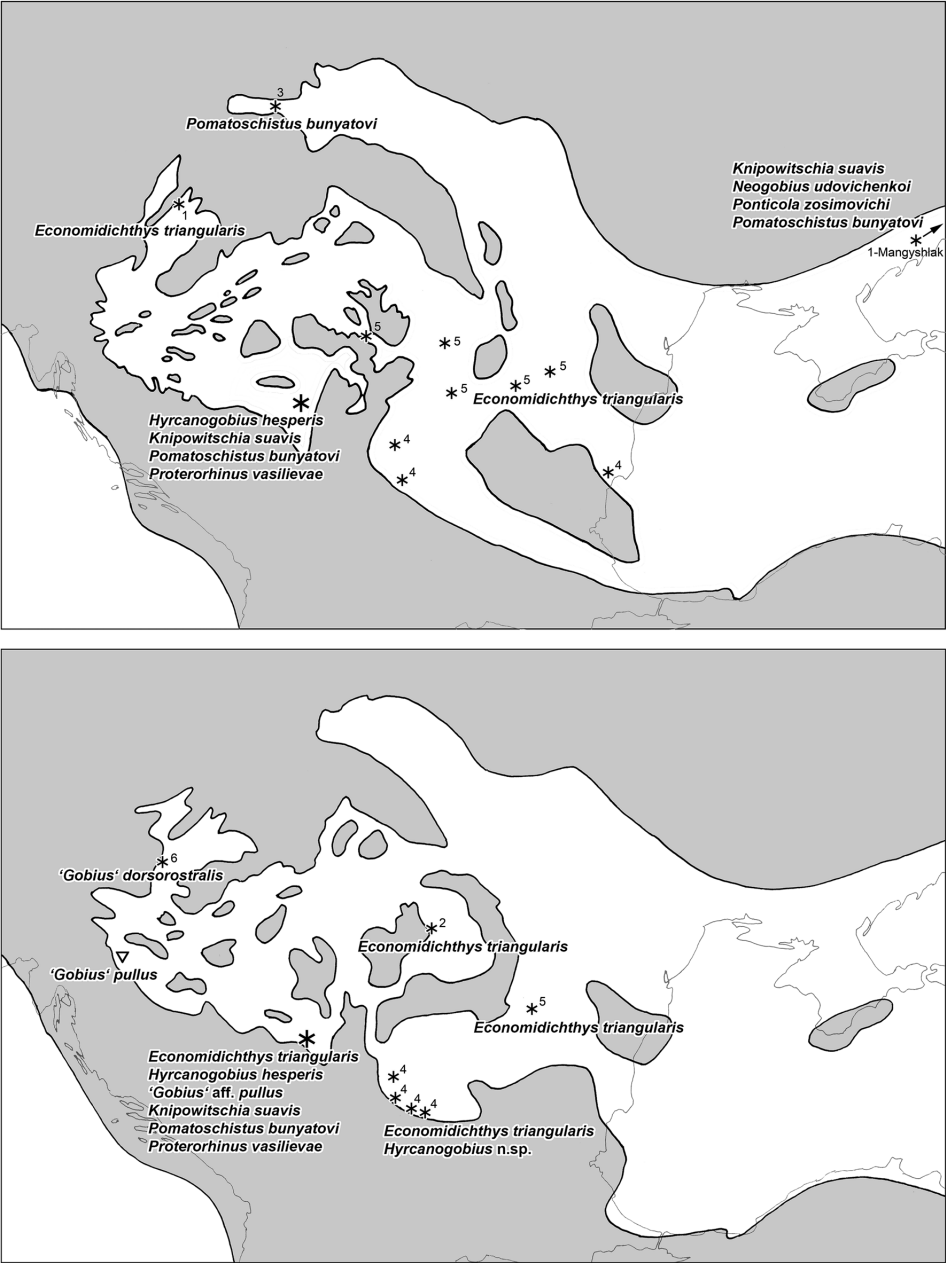
*1* Distribution of otolith-based sand goby and Ponto-Caspian goby species in the central and western part of Eastern Paratethys during late Badenian/Konkian. *2* Distribution of otolith-based sand goby and Ponto-Caspian goby species in the central and western part of Eastern Paratethys during Sarmatian s.s./Volhynian. Paleogeographic reconstructions overlain on recent geography and based on Rögl (1998), Kovač et al. (2007) and Lukeneder et al. (2011). Otolith locations marked by *small asterisk* and number = *1* Bratishko et al. (2015; including reference to Schubert 1906), *2* Brzobohaty and Stancu (1974), *3* Smigielska (1966), *4* Strashimirov (1981a, b, 1984, 1985a, b; selected and adjusted according to ongoing review), *5* Weiler (1943, 1949, 1950; adjusted after Nolf 2013 and Bratishko et al. 2015), and *6* Schwarzhans (2010); *large asterisk* this work; *open triangle* skeleton with otolith in situ from Kramberger-Gorjanovic (1883)

The late Badenian/Konkian and the Sarmatian otolith associations of the Central Paratethys and the Eastern Paratethys are showing a number of shared species, particularly in gadids and gobiids, and following ongoing research and review of earlier publications, this correlation is expected to increase. Shared species are in Gadidae *Palimphemus minusculoides* and *Paratrisopterus insectus* (Weiler 1943) and in Gobiidae *Economidichthys triangularis*, *Knipowitschia suavis*, and *Pomatoschistus bunyatovi* (which is already known in the Paratethys since early Badenian). However, there also appears to be replacement species in some gobiid genera from the eastern part of the Central Paratethys to the western part of the Eastern Paratethys. For instance, *Hyrcanogobius hesperis* appears to be replaced by another, undescribed species of *Hyrcanogobius* in the Dacian Basin. The current status of gobiid species distribution across the Central to Eastern Paratethys interface is depicted in Fig. 10.

### Biostratigraphic observations

Table 1 summarizes the stratigraphic distribution of the otolith species observed at Slanci and in the Barajevo wells. Evidently, the major faunal break between early and late Badenian is well depicted. Not a single species is shared across the boundary, but we argue that this primarily reflects an environmental change. The late Badenian and Sarmatian interval in contrast does not show any significant faunal changes that could be considered as of stratigraphic relevance, with the possibly exception of *Knipowitschia suavis* not extending upward of the early Volhynian. However, one must also acknowledge that the obtained otolith specimens from the Barajevo wells are few and may not be considered as statistically relevant for a stratigraphic evaluation.
